# Development of a preoperative accuracy prediction model using machine learning for implant placement in static-guided surgery: retrospective observational study

**DOI:** 10.1186/s40729-026-00696-0

**Published:** 2026-06-22

**Authors:** Takuya Mino, Yurina Matsuoka, Ken’ichi Morooka, Kana Tokumoto, Hiroaki Shimizu, Yoko Kurosaki, Aya Kimura-Ono, Hiromitsu Kishimoto, Takuo Kuboki, Kenji Maekawa

**Affiliations:** 1 Department of Removable Prosthodontics and Occlusion, School of Dentistry, Osaka Dental University, 1-5-17 Otemae, Chuo-ku, Osaka, 540-0008 Japan; 2 Department of Semiconductor, Computer Science and Applied Mathematics, Graduate School of Science and Technology, Kumamoto University, 2-39-1 Kurokami, Chuo-ku, Kumamoto, 860-8555 Japan; 3 Division of Biomedical Engineering, Faculty of Advanced Science and Technology, Kumamoto University, 2-39-1 Kurokami, Chuo-ku, Kumamoto, 860-8555 Japan; 4 Department of Oral and Maxillofacial Surgery, School of Medicine, Hyogo Medical University, 1-1 Mukogawacho, Nishinomiya, 663-8501 Japan; 5 Shimizu Dental Clinic, 7-1-9 Kamisawa-dori, Hyogo-ku, Kobe, 652- 0046 Japan; 6 Center for Innovative Clinical Medicine, Okayama University Hospital, 2-5- 1 Shikata-cho, Okayama, 700-8525 Japan; 7 Department of Oral Rehabilitation and Regenerative Medicine, Okayama University Faculty of Medicine, Dentistry and Pharmaceutical Sciences, 2-5-1 Shikata-cho, Okayama, 700-8525 Japan

**Keywords:** Implant placement, Accuracy, Surgical guide, Machine learning, Prediction algorithms, Decision trees, Support vector machine, Sensitivity and specificity

## Abstract

**Purpose:**

This study aimed to develop a machine learning model capable of preoperatively predicting three-dimensional implant placement errors at the implant apex in static-guided surgery and to identify the clinical features associated with placement accuracy.

**Methods:**

Clinical data partially derived from a previous observational study were analyzed. In total, 181 patients and 480 implants placed using fully static-guided surgery were included in this study. The outcome variable was defined as three-dimensional implant placement error at the implant apex relative to the preoperative simulation, dichotomized as less than 0.5 mm or ≥ 0.5 mm. Twenty-one clinical and radiographic factors previously suggested to influence the placement accuracy were used as explanatory variables. The feature importance was evaluated using three gradient boosting decision tree models. Furthermore, a stacking model combining multiple classifiers was constructed, and the classification performance was assessed using ten-fold cross-validation.

**Results:**

The feature importance analysis identified 12 features associated with implant placement errors. The stacking model demonstrated superior classification performance compared to individual classifiers. The true positive rate was 0.73, false negative rate was 0.27, false positive rate was 0.14, and true negative rate was 0.86.

**Conclusions:**

The proposed stacking model correctly classified 86% of cases with implant placement error less than 0.5 mm and 73% of cases with implant placement error of ≥ 0.5 mm. These findings suggest that the proposed model may support the preoperative evaluation of implant placement accuracy in static-guided surgeries.

**Supplementary Information:**

The online version contains supplementary material available at https://doi.org/10.1186/s40729-026-00696-0.

## Background

Oral implant therapy is a widely established predictive treatment modality. In recent years, computer-assisted implant placement using static-guided surgery (SGP) has been increasingly adopted in clinical practice to improve the reproducibility of implant positioning based on preoperative planning. A systematic review demonstrated that SGP generally provides a higher accuracy than freehand techniques [1]. However, clinically relevant three-dimensional deviations between planned and placed implant positions have been reported, even when SGP is used [2].

Such implant placement errors may compromise the spatial relationship between implants and adjacent anatomical structures, including the inferior alveolar nerve and maxillary sinus, and may also affect the prosthetic design and restorative outcomes. These deviations have been reported to influence the treatment safety and long-term prognosis in guided implant surgeries [3]. Therefore, improving the predictability of implant placement remains an important clinical challenge.

With advances in digital dentistry, artificial intelligence (AI) and machine learning techniques have been increasingly applied in oral implantology. Previous studies reported AI-based approaches for implant planning support and automated or optimized implant planning [4–6]. In addition, deep-learning-based methods have been developed for three-dimensional planning support, including automated extraction of edentulous regions and estimation of implant positions from cone-beam computed tomography (CBCT) images [7–9]. Furthermore, AI approaches have been applied to preoperative feasibility assessment and prognosis prediction in implant dentistry [10, 11].

Despite this growing body of literature, limited attention has been directed toward the preoperative prediction of implant placement accuracy at the individual case level. From a clinical perspective, the ability to predict placement errors before surgery allows clinicians to reconsider treatment strategies during the simulation phase, such as modifying implant positioning, selecting alternative surgical guide designs, and adjusting surgical protocols. This approach may contribute to improved placement accuracy, the avoidance of unexpected complications, and safer implant treatments.

Therefore, this observational study aimed to develop a machine learning-based model capable of preoperatively predicting three-dimensional implant placement errors at the implant apex using clinical data obtained from SGP implant surgery. In addition, because machine learning models often incorporate complex nonlinear relationships and interactions that are difficult to interpret [12], this study also employed feature importance analysis to identify the clinical and radiographic factors associated with implant placement errors.

## Methods

### Dataset

This observational study was conducted in accordance with the Declaration of Helsinki and approved by the Clinical Research Review Committee of Okayama University (Approval No. Ken 2312−002).

This study utilized a portion of the dataset collected in a previous observational study [2]. The inclusion criteria comprised all patients who underwent fully guided implant placement using SGP at a private dental clinic (Shimizu Dental Clinic) between March 1, 2014, and March 1, 2018. All surgeries were performed at a single site by one experienced operator to ensure procedural consistency. Cases were excluded if informed consent for participation in the study was not obtained or if automatic matching between preoperative and postoperative computed tomography (CT) images could not be successfully performed.

A total of 181 patients (480 implants) were included in the final analysis. Clinical and radiographic variables that had been suggested by previous studies or clinical experience as potentially influencing the accuracy of SGP implant placement were extracted from the clinical records. These variables included age at implant placement surgery, sex, type of SGP, number of remaining teeth in the treated jaw, number of coronal teeth, number of restored teeth associated with imaging artifacts, number of placed implants, type of dentition defect, implant location (maxillary or mandibular, anterior or posterior), implant length, implant width, shape of the implant body, shape of the implant drill, distance between the guide sleeve bottom and bone surface, presence or absence of bone augmentation, number of fixation pins, and limited mouth opening.

In addition, the alveolar bone quality, mesiodistal bone surface inclination, and buccolingual bone surface inclination were assessed using preoperative CT images.

Following automatic matching of the pre- and postoperative images, the three-dimensional deviation of the implant apex relative to the preoperative simulation was measured using the simulation software’s distance measurement tool and defined as the implant placement error. No data were missing for any variables included in the final analysis.

### Identification of important features associated with implant placement error using gradient boosting decision trees

To identify clinical and radiographic features associated with implant placement error in SGP, three gradient boosting decision tree models were employed; Light Gradient Boosting Machine (LGB) [13], eXtreme Gradient Boosting (XGB) [14], and CatBoost (CB) [15]. These models were selected because of their ability to capture nonlinear relationships and complex interactions among explanatory variables.

The objective variable, implant placement error, was defined as the three-dimensional deviation of the implant apex relative to the preoperative simulation and dichotomized as less than 0.5 mm [0] or ≥ 0.5 mm [1]. The 0.5-mm threshold was selected based primarily on discussions among implant specialists experienced in static-guided implant surgery regarding the degree of deviation from the preoperative simulation that would generally be considered clinically acceptable in daily practice. All clinical and radiographic variables described in Sect. 2.1 were used as explanatory variables.

The analytical dataset was divided into 10 subsets. Two subsets were used for hyperparameter tuning and the remaining eight subsets were used for 8-fold cross-validation. During the cross-validation, one subset was assigned as the test dataset, one subset as the validation dataset, and the remaining six subsets as the training dataset. This process was repeated eight times, with each subset serving once as the test dataset.

The feature importance was evaluated using multiple metrics derived from each gradient boosting decision tree model. Eight evaluation metrics were employed to quantify the feature importance: gain, which represents the improvement in the loss function achieved by a split in the decision tree; split, which indicates the number of times a feature is used for splitting across the entire model; cover, which reflects the total number of samples affected by a particular split; total gain, which is the sum of gain values for all splits involving a given feature; total cover, which is the sum of cover values for all splits involving a given feature; and prediction value change, which represents the magnitude of change in the model output associated with variations in a specific feature. For LGB, the gain and split were calculated [16]. For XGB, gain, cover, total gain, total cover, and weight were calculated [17]. For the CB, prediction value changes were used [18]. The top five ranked features were identified for each metric. Because no standardized criterion exists for determining the number of important features in feature importance analysis, features that appeared at least once among the top five across all evaluation metrics were selected as important features associated with implant placement error, as described in previous studies [19–21].

### Construction of the machine learning stacking model for predicting implant placement error in SGP implant surgery (Proposed method)

To develop a prediction model for implant placement errors in SGP, a stacking-based machine learning approach was employed. All clinical and radiographic variables described in Sect. 2.1 were used as explanatory variables; the objective variable was defined as the binarized three-dimensional implant placement error at the implant apex, classified as less than 0.5 mm [0] or ≥ 0.5 mm [1].

In addition to the three gradient boosting decision trees, support vector machine (SVM) [22] and random forest (RF) [23] classifiers were included to complement the characteristics and limitations of the three decision tree techniques. The stacking model consists of five base classifiers: LGB, two CB models with different parameter settings, SVM, and RF. The prediction outputs of these base classifiers are subsequently integrated using the CB model as a meta-learner.

**Fig. 1:**
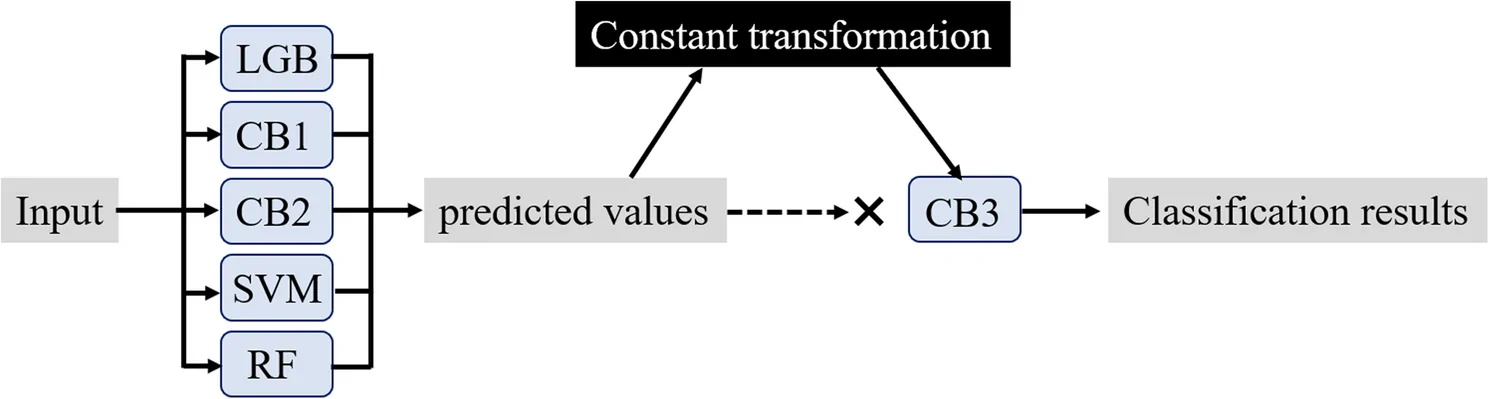
Schematic illustration of the stacking-based machine learning model. Prediction outputs generated by the base classifiers were transformed into categorical constants and subsequently used as input variables for the CatBoost meta-learner. The “×” symbol indicates that the original continuous prediction outputs were not directly input into the meta-learner. Instead, the outputs were transformed into categorical constants using predefined thresholds. Solid arrows indicate the analytical flow adopted in the present study, whereas dashed arrows indicate the conventional direct-input flow that was not used in the proposed model

For each base classifier, the output continuous value representing the confidence that the implant placement error was ≥ 0.5 mm was obtained. These continuous prediction values were transformed into categorical constants according to the predefined thresholds listed in Table 1. This constant transformation is applied to facilitate effective learning by the CB meta-learner, which is particularly suitable for handling categorical variables. A schematic of the stacking procedure is shown in Fig. 1.

**Table 1:** Criteria for constant transformation of prediction outputs using Youden’s J statistic

Criteria	Transformed constant value
X < J −0.3	1
J −0.3 ≤ X < J −0.2	5
J −0.2 ≤ X < J −0.1	10
J −0.1 ≤ X < J	15
J ≤ X	30

X: predicted probability of class 1

J: Youden’s J statistic

### Machine learning analysis

The analytical procedure for constructing and evaluating the machine learning stacking model is as follows: First, the entire dataset was divided into 10 subsets. Cross-validation was performed at the implant level. Therefore, implants originating from the same patient or surgical guide could potentially be included across different folds. One subset was assigned as the test dataset and the remaining nine subsets were used for model development. For each of the five base classifiers described in Sect. 2.3, a threefold cross-validation was performed using nine subsets, with one subset used for validation and the remaining subsets used for training. From each base classifier, a continuous output value representing the confidence that the implant placement error was ≥ 0.5 mm was obtained for each implant.

These output values were then transformed into categorical constants according to predefined thresholds determined using Youden’s J statistic, which is defined as the true positive rate minus the false positive rate [24]. The transformed outputs of the five base classifiers are subsequently used as input variables for the CB meta-learner. After constant transformation, 90% of the transformed dataset was used as training data and 10% as validation data. 10-fold cross validation was performed to train the meta-learner. The trained stacking model was subsequently applied to the test dataset to generate the classification results.

This entire procedure was repeated ten times so that each subset was used once as the test dataset. The classification performance was evaluated using the F1-score, which is defined as the harmonic mean of precision and recall. In addition, precision, recall, and accuracy were calculated to provide a more comprehensive assessment of model performance. For comparison, a 10-fold cross-validation was conducted for each base classifier, and the corresponding F1-scores were calculated. Prior to classifier construction, scatter plot assessment of the relationships between implant placement error and explanatory variables was performed. Three implants exhibiting extreme outlier values in three-dimensional implant placement error (> 2.5 mm) were identified as clearly separated from the overall distribution and were excluded from the classification analysis to reduce the influence of atypical deviations on model training and evaluation.

## Results

### Dataset

The feature importance analysis included all 480 implants obtained from 181 cases treated using static-guided surgery. The clinical and radiographic characteristics of the study population and implants are summarized in Table 2. For construction and evaluation of the classification model, three implants exhibiting extreme outlier values in three-dimensional implant placement error (> 2.5 mm) were excluded. Consequently, the classification analysis was conducted using 477 implants. The dataset included a wide range of patient-, implant-, and surgical guide-related variables, reflecting the diverse clinical conditions encountered during SGP.

**Table 2:** Clinical and radiographic characteristics of patients and implants included in the study

Factors related to each surgical guide	*n* = 181
Sex (male/female; number of subjects)	64/117
Age at implant placement surgery (years; mean ± SD)	61.8 ± 11.4
Limited mouth opening (with/without; number of subjects)	19/162
Type of SGP (SCT/DCT/MSCT; number of subjects)	27/88/66
Number of remaining teeth (mean ± SD)	8.4 ± 4.0
Number of coronal teeth (mean ± SD)	9.0 ± 4.1
Number of restored teeth associated with artifacts (mean ± SD)	3.9 ± 3.2
Number of placed implants (mean ± SD)	2.9 ± 1.9
Dentition defect type (Kennedy Classification Class I/II/III/IV/complete edentulism; number of subjects)	28/54/60/17/22
Number of fixation pins (mean ± SD)	4.1 ± 2.3

Factors related to each implant body	*n* = 480
Shape of implant body (straight/tapered; number of implants)	62/418
Implant width (NP/RP/WP; number of implants)	149/290/41
Implant length (mm; mean ± SD)	11.7 ± 2.2
Shape of implant drill (straight/tapered; number of implants)	150/330
Implant location (anterior/posterior; number of implants)	132/348
Implant location (maxillary/mandibular; number of implants)	261/219
Bone augmentation (with/without; number of implants)	35/445
Alveolar bone quality (1/2/3/4; number of implants)	15/114/235/116
Distance between guide sleeve bottom to bone surface (4.0/5.5/6.0 mm; number of implants)	105/268/107
Buccolingual bone surface inclination (0–90 degree; mean ± SD)	67.2 ± 17.5
Mesiodistal bone surface inclination (0–90 degree; mean ± SD)	73.8 ± 14.3
Implant placement error (mm; mean ± SD, < 0.5 mm/≥0.5 mm; number of implants)	0.98 ± 0.52, 92/388

*CT* Computed tomography, *SCT* Single CT scan, *DCT* Double CT scan, *MSCT* Modified single CT scan, *NP* Narrow platform, *RP* Regular platform, *WP* Wide platform, *SD* Standard deviation

### Important features associated with implant placement error in SGP implant surgery

**Fig. 2:**
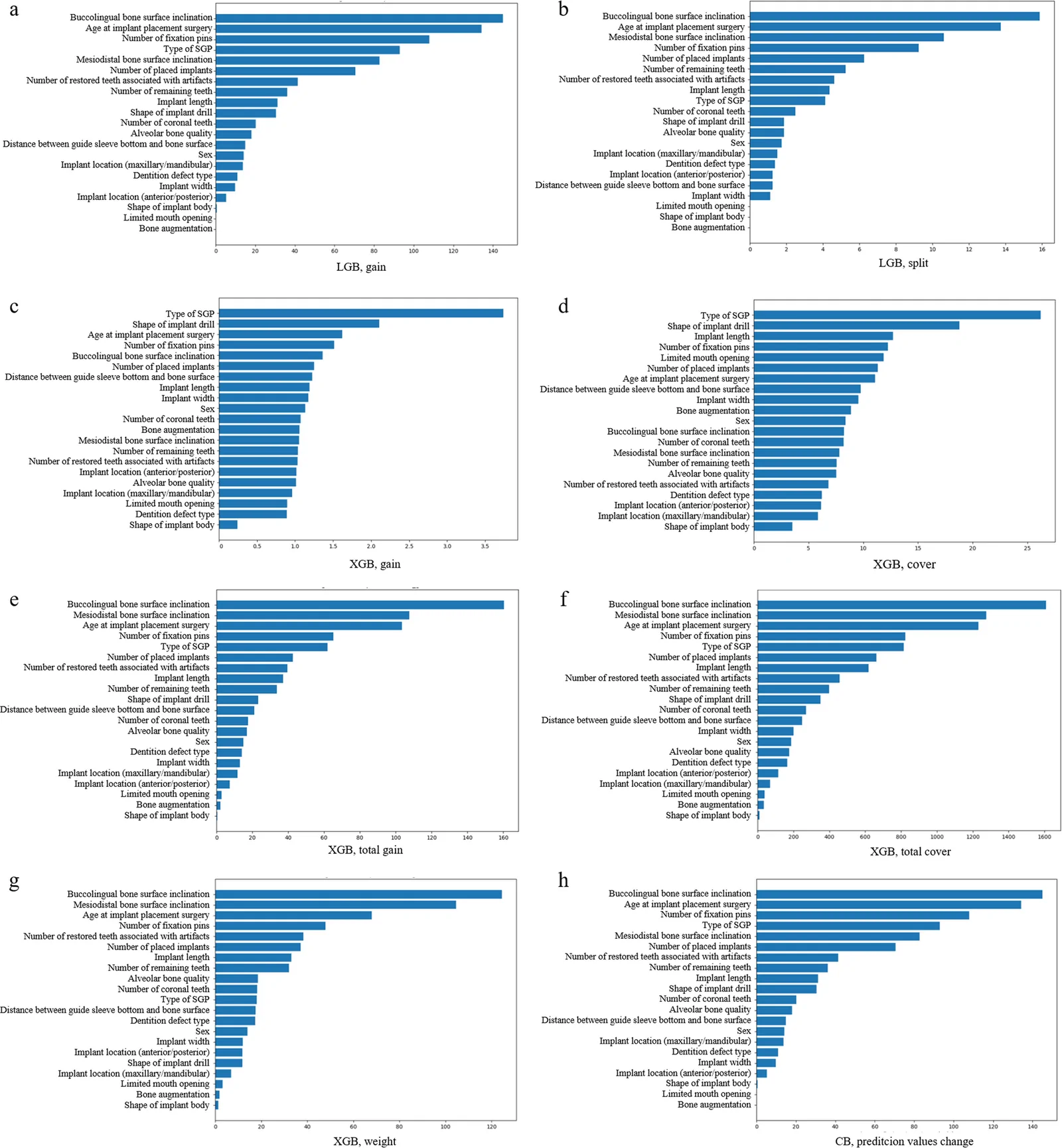
Important features associated with implant placement error identified using gradient boosting and feature importance metrics. **a** Light Gradient Boosting using gain. **b** Light Gradient Boosting using split. **c** eXtreme Gradient Boosting using gain. **d** eXtreme Gradient Boosting using cover. **e** eXtreme Gradient Boosting using total gain. **f** eXtreme Gradient Boosting using total cover. **g** eXtreme Gradient Boosting using weight. **h** CatBoost using prediction values change

The results of the feature importance analysis performed using the three gradient boosting decision tree models and eight evaluation metrics are shown in Fig. 2. Across all the analyses, a total of 12 features were identified as important, having appeared at least once among the top five ranked features for any evaluation metric.

When ranked by the frequency of selection across the eight analyses, age at implant placement surgery, buccolingual bone surface inclination, and number of fixation pins were the most frequently identified features. This was followed by the SGP and mesiodistal bone surface inclination. Additional features identified as important included the number of placed implants, shape of the implant drill, presence of limited mouth opening, number of remaining teeth, number of restored teeth associated with artifacts, implant length, and the distance between the guide sleeve bottom and the bone surface.

### Classification performance of the machine learning stacking model for predicting implant placement error

The classification performances of the proposed stacking-based machine learning model and individual classifiers are summarized in Table 3. Additional performance metrics, including precision, recall, and accuracy, are provided in Supplementary Tables S1–S3. The stacking model achieved a higher mean F1-score than each individual classifiers across ten-fold cross validation.

**Table 3:** Classification performance of the stacking model and individual classifiers based on the F1-score

Test dataset	LGB	CB1	CB2	SVM	RF	Proposed method
1	0.68	0.73	0.81	**0.83**	0.67	0.68
2	0.78	0.87	0.67	0.87	0.85	**0.90**
3	0.84	0.77	**0.90**	0.76	0.80	0.81
4	0.42	0.55	**0.86**	0.75	0.61	0.73
5	0.69	0.73	**0.94**	0.88	0.79	0.91
6	0.73	0.48	0.59	0.51	0.59	**0.91**
7	0.77	0.55	0.52	**0.86**	0.73	0.69
8	0.92	**0.95**	0.86	0.85	0.94	0.79
9	0.80	0.70	0.85	0.71	0.81	**0.86**
10	0.87	0.89	**0.95**	0.92	0.82	0.95
mean	0.75	0.72	0.79	0.80	0.76	**0.82**
variance	0.017	0.022	0.02	0.013	0.011	0.009

Bold numbers represent the highest F1-score in each test dataset

*F1-score* Harmonic mean of precision and recall, *LGB* Light Gradient Boosting Machine, *CB* CatBoost, *SVM* Support Vector Machine, *RF* Random Forest

The confusion matrices obtained from the classification results of the stacking method are listed in Table 4. Using the proposed model, 86% of cases with implant placement error less than 0.5 mm were correctly classified as negative cases, whereas 73% of cases with implant placement error of ≥ 0.5 mm were correctly classified as positive cases. The corresponding false positive rate was 0.14, and false negative rate was 0.27.

**Table 4:** Confusion matrix of the stacking-based machine learning model for classification of implant placement error

	Predicted class: 1	Predicted class: 2
True class: 1	0.73(True Positive Rate)	0.27(False Negative Rate)
True class: 2	0.14(False Positive Rate)	0.86(True Negative Rate)

Positive indicates implant placement error ≥ 0.5 mm, and negative indicates implant placement error < 0.5 mm

Balanced Accuracy (mean of true positive rate and true negative rate) = 0.80

## Discussion

This study investigated the clinical and radiographic factors associated with implant placement errors in SGP implant surgery using machine learning techniques, and developed a stacking-based classifier for the preoperative prediction of implant placement errors. Feature importance analysis identified multiple factors related to patient characteristics, anatomical conditions, and surgical procedures, whereas the proposed ensemble model demonstrated stable classification performance. The clinical and methodological implications of these findings are discussed in the following sections.

### Important features associated with implant placement error in SGP implant surgery

In this study, gradient boosting decision tree-based feature importance analysis identified 12 clinical and radiographic factors associated with three-dimensional implant placement errors in SGP. Among these, age at implant placement, buccolingual bone surface inclination, and number of fixation pins were the most frequently selected features across multiple evaluation metrics.

Age at implant placement surgery has previously been reported as a factor associated with implant placement accuracy, potentially reflecting age-related changes in bone quality, mucosal resilience, and functional conditions, such as mouth opening [2]. Although age itself is not a direct surgical factor, it may serve as a surrogate marker for multiple anatomical and functional characteristics that influence seating stability and positioning of surgical guides.

Buccolingual and mesiodistal bone surface inclinations have also been consistently identified as important features. These anatomical characteristics may affect the stability of the surgical guide seated on the alveolar ridge and influence drill trajectory control during osteotomy preparation. In cases of pronounced ridge inclination, even minor discrepancies in guide positioning may result in clinically relevant apical deviations despite the use of static guidance [3].

The number of fixation pins was also frequently identified as an important predictor associated with implant placement error. Fixation pins are intended to enhance guide stability and reduce micromovements during drilling. However, the present findings suggest that the fixation strategy itself may contribute to placement errors, possibly reflecting differences in clinical complexity, anatomical constraints, or guide design, rather than a simple linear relationship between pin number and placement accuracy [2].

Several additional factors, including the type of static-guided surgery, number of placed implants, shape of the implant drill, limited mouth opening, number of remaining teeth, presence of imaging artifacts, implant length, and the distance between the guide sleeve bottom and the bone surface, were also identified as important features. These variables may influence implant placement accuracy through their effects on guide design, surgical access, imaging precision, and the reproducibility of preoperative planning and simulation [25].

Overall, these findings indicate that the implant placement accuracy in static-guided surgery is influenced by a combination of patient, anatomical, and procedural factors. The ability of machine learning models to integrate such complex and potentially nonlinear interactions may provide additional insights into the factors affecting implant placement errors beyond conventional linear analytical approaches.

### Classification performance of the machine learning stacking model for predicting implant placement error in SGP implant surgery

In the present study, the stacking-based machine learning model demonstrated a higher classification performance than the individual classifiers. Stacking is an ensemble learning approach designed to integrate the complementary strengths of multiple algorithms. Previous studies have shown that this strategy can improve predictive performance while reducing the risk of overfitting compared to single-model approaches [26–29].

A characteristic feature of the proposed model is the use of constant transformation based on Youden’s J-statistics. In the first layer, each base classifier generated a continuous output, representing confidence that the implant placement error was ≥ 0.5 mm. These outputs are converted into categorical constants using thresholds determined by Youden’s J statistic, which is widely used to optimize the balance between sensitivity and specificity in binary classification problems [24]. The transformed categorical outputs are then used as input variables for the CB meta-learner, which is particularly effective for handling categorical features [15]. This two-step strategy may have contributed to the stable classification performance observed in this study.

From a clinical perspective, the preoperative classification of implant placement error risk may support decision-making during the treatment planning phase. Cases predicted to have a higher risk of implant placement error may warrant additional consideration during the preoperative simulation phase. Potential modifications may include reconsideration of implant positioning, adjustment of the surgical guide design or fixation strategy, selection of alternative drilling protocols, or the use of additional safety margins in anatomically restricted areas. In some cases, clinicians may also consider alternative treatment approaches when a higher risk of clinically relevant deviation is anticipated. Importantly, the proposed model is intended to support clinical judgment rather than replace it, which is consistent with current perspectives on the role of machine learning as a decision support tool in clinical practice [12].

### Limitations

This study has several limitations. First, the clinical data were obtained from a single implant specialist with extensive experience in SGP implant surgeries. Consequently, the findings may not be directly generalizable to clinicians with different levels of experience or to institutions with varying surgical protocols. In addition, operator-related factors such as surgical skill, decision-making, and learning curves could not be evaluated separately in the present analysis.

Second, the dataset was retrospectively collected from a single clinical site. Although this approach ensured a high degree of procedural consistency and minimized the variability related to the surgical technique, it may have limited the external validity of the proposed model. Future studies incorporating multicenter data and multiple operators are warranted to further validate the robustness and generalizability of these findings.

Third, the outcome variable was dichotomized using a threshold of 0.5 mm for three-dimensional implant placement error at the implant apex. While this threshold was selected based on clinical considerations and previous studies, dichotomization may have resulted in some loss of information compared to continuous outcome modeling.

Finally, although the proposed model demonstrated stable classification performance, the relatively limited sample size and exclusion of extreme outlier cases may have influenced the model behavior. Prospective validation using independent datasets is necessary before clinical implementation. In addition, because cross-validation was performed at the implant level rather than at the patient or surgical-guide level, implants derived from the same patient could potentially have been distributed across different folds. This may have introduced dependency among samples and resulted in optimistic estimates of model performance. Future studies using grouped cross-validation and external validation datasets are warranted to further evaluate the robustness and generalizability of the proposed model.

## Conclusions

Within the limitations of this study, multiple clinical and radiographic factors associated with three-dimensional implant placement errors in static-guided surgery were identified using machine learning-based feature importance analysis. These findings indicate that implant placement accuracy was associated with a combination of patient-related, anatomical, and procedural factors, rather than a single determinant.

Furthermore, a stacking-based machine learning model integrating multiple classifiers demonstrated a stable predictive performance for the preoperative classification of implant placement errors at the implant apex. The proposed approach correctly classified the majority of cases with both low and high placement errors, suggesting its potential utility as a decision-support tool during the treatment planning phase.

Taken together, these results suggest that machine learning-based preoperative evaluation may support a more informed planning of static-guided implant surgery and contribute to safer and more predictable implant treatments. Further validation using prospective and multicenter datasets is required before clinical implementation.

## Supplementary Information

## Data Availability

The datasets generated and analyzed during the current study are available from the corresponding author on reasonable request.

## Abbreviations

AI: Artificial intelligence
CB: CatBoost
CBCT: Cone-beam computed tomography
CT: Computed tomography
DCT: Double Computed Tomography
F1-score: Harmonic mean of precision and recall
LGB: Light Gradient Boosting Machine
ML: Machine learning
MSCT: Modified single computed tomography
NP: Narrow platform
RF: Random forest
RP: Regular platform
SCT: Single computed tomography
SD: Standard deviation
SGP: Static-guided surgery
SVM: Support vector machine
WP: Wide platform
XGB: EXtreme Gradient Boosting
