# Supplementary material for: Development of a preoperative accuracy prediction model using machine learning for implant placement in static-guided surgery: retrospective observational study
Source: Int J Implant Dent. 2026 Jun 22;12:44. doi: 10.1186/s40729-026-00696-0 (PMC13538333; doi:10.1186/s40729-026-00696-0)
Supplement: Supplementary file 1 [file 40729_2026_696_MOESM1_ESM.docx]

| Supplementary Table S1. Classification performance of the stacking model and individual classifiers based on precision. | | | | | | |
| --- | --- | --- | --- | --- | --- | --- |
| Test dataset | LGB | CB1 | CB2 | SVM | RF | Proposed method |
| 1 | **1.00** | 0.96 | 0.93 | 0.91 | 0.95 | **1.00** |
| 2 | 0.89 | 0.89 | **1.00** | 0.81 | 0.91 | 0.94 |
| 3 | 0.93 | **1.00** | 0.87 | 0.86 | 0.87 | 0.93 |
| 4 | 0.92 | **1.00** | 0.89 | 0.90 | 0.95 | 0.92 |
| 5 | **1.00** | **1.00** | 0.97 | 0.94 | **1.00** | 0.97 |
| 6 | 0.87 | **1.00** | **1.00** | **1.00** | **1.00** | 0.97 |
| 7 | 0.86 | **0.93** | **0.93** | 0.84 | 0.85 | 0.91 |
| 8 | **1.00** | **1.00** | **1.00** | 0.94 | 0.97 | **1.00** |
| 9 | 0.90 | 0.92 | 0.89 | **0.96** | 0.88 | 0.94 |
| 10 | **1.00** | **1.00** | 0.97 | 0.97 | **1.00** | **1.00** |
| mean | 0.93 | **0.97** | 0.95 | 0.91 | **0.97** | 0.96 |
| variance | 0.003 | 0.0017 | 0.0023 | 0.0034 | 0.003 | 0.0011 |
| Bold numbers represent the highest Precision in each test dataset. | | | | |  |  |
| LGB: Light Gradient Boosting Machine, CB: CatBoost | | | |  |  |  |
| SVM: Support Vector Machine, RF: Random Forest | | | |  |  |  |

| Supplementary Table S2. Classification performance of the stacking model and individual classifiers based on recall. | | | | | | |
| --- | --- | --- | --- | --- | --- | --- |
| Test dataset | LGB | CB1 | CB2 | SVM | RF | Proposed method |
| 1 | 0.51 | 0.59 | 0.72 | **0.77** | 0.51 | 0.51 |
| 2 | 0.69 | 0.86 | 0.50 | **0.94** | 0.81 | 0.86 |
| 3 | 0.77 | 0.63 | **0.94** | 0.69 | 0.74 | 0.71 |
| 4 | 0.28 | 0.38 | **0.83** | 0.65 | 0.45 | 0.60 |
| 5 | 0.53 | 0.58 | **0.90** | 0.83 | 0.65 | 0.85 |
| 6 | 0.63 | 0.32 | 0.41 | 0.34 | 0.41 | **0.85** |
| 7 | 0.69 | 0.39 | 0.36 | **0.89** | 0.64 | 0.56 |
| 8 | 0.85 | **0.90** | 0.75 | 0.78 | **0.90** | 0.65 |
| 9 | 0.72 | 0.56 | **0.82** | 0.56 | 0.74 | 0.79 |
| 10 | 0.77 | 0.79 | **0.92** | 0.87 | 0.69 | 0.90 |
| mean | 0.64 | 0.60 | 0.72 | **0.73** | 0.66 | **0.73** |
| variance | 0.0253 | 0.0374 | 0.0416 | 0.0291 | 0.022 | 0.018 |
| Bold numbers represent the highest Recall in each test dataset. | | | | |  |  |
| LGB: Light Gradient Boosting Machine, CB: CatBoost | | | |  |  |  |
| SVM: Support Vector Machine, RF: Random Forest | | | |  |  |  |

| Supplementary Table S3. Classification performance of the stacking model and individual classifiers based on accuracy. | | | | | | |
| --- | --- | --- | --- | --- | --- | --- |
| Test dataset | LGB | CB1 | CB2 | SVM | RF | Proposed method |
| 1 | 0.60 | 0.64 | 0.72 | **0.74** | 0.57 | 0.60 |
| 2 | 0.71 | 0.81 | 0.63 | 0.79 | 0.79 | **0.85** |
| 3 | 0.79 | 0.72 | **0.85** | 0.68 | 0.72 | 0.74 |
| 4 | 0.38 | 0.48 | **0.77** | 0.65 | 0.52 | 0.63 |
| 5 | 0.60 | 0.65 | **0.90** | 0.81 | 0.71 | 0.85 |
| 6 | 0.60 | 0.42 | 0.50 | 0.44 | 0.50 | **0.85** |
| 7 | 0.69 | 0.52 | 0.50 | **0.79** | 0.65 | 0.63 |
| 8 | 0.87 | **0.91** | 0.79 | 0.77 | 0.89 | 0.70 |
| 9 | 0.71 | 0.60 | 0.77 | 0.63 | 0.71 | **0.79** |
| 10 | 0.80 | 0.82 | 0.91 | 0.86 | 0.73 | **0.91** |
| mean | 0.67 | 0.66 | 0.73 | 0.72 | 0.68 | **0.76** |
| variance | 0.0175 | 0.0232 | 0.0197 | 0.0138 | 0.0134 | 0.0117 |
| Bold numbers represent the highest Accuracy in each test dataset. | | | | |  |  |
| LGB: Light Gradient Boosting Machine, CB: CatBoost | | | |  |  |  |
| SVM: Support Vector Machine, RF: Random Forest | | | |  |  |  |
